# Effects of Different Antidiabetic Medications on Endothelial Glycocalyx, Myocardial Function, and Vascular Function in Type 2 Diabetic Patients: One Year Follow–Up Study

**DOI:** 10.3390/jcm8070983

**Published:** 2019-07-05

**Authors:** Vaia Lambadiari, George Pavlidis, Foteini Kousathana, Eirini Maratou, Dimitrios Georgiou, Ioanna Andreadou, Aikaterini Kountouri, Maria Varoudi, Konstantinos Balampanis, John Parissis, Helen Triantafyllidi, Konstantinos Katogiannis, Dionysia Birba, John Lekakis, George Dimitriadis, Ignatios Ikonomidis

**Affiliations:** 1 2nd Department of Internal Medicine, Research Unit and Diabetes Centre, Attikon Hospital, National and Kapodistrian University of Athens, Medical School, 12462 Athens, Greece; 2 Laboratory of Clinical Biochemistry, Attikon Hospital, National and Kapodistrian University of Athens, Medical School, 12462 Athens, Greece; 3 Department of Pharmaceutical Chemistry, National and Kapodistrian University of Athens, School of Pharmacy, 15741 Athens, Greece; 4 2nd Department of Cardiology, Attikon Hospital, National and Kapodistrian University of Athens, Medical School, 12462 Athens, Greece

**Keywords:** glycaemic control, endothelial glycocalyx, arterial stiffness, left ventricular function

## Abstract

Background: Poor glycaemic control affects myocardial function. We investigated changes in endothelial function and left ventricular (LV) myocardial deformation in poorly controlled type 2 diabetics before and after glycaemic control intensification. Methods: In 100 poorly-controlled diabetic patients (age: 51 ± 12 years), we measured at baseline and at 12 months after intensified glycaemic control: (a) Pulse wave velocity (PWV, Complior); (b) flow-mediated dilatation (FMD, %) of the brachial artery; (c) perfused boundary region (PBR) of the sublingual arterial micro-vessels (side-view dark-field imaging, Glycocheck); (d) LV global longitudinal strain (GLS), peak twisting (pTw), peak twisting velocity (pTwVel), and peak untwisting velocity (pUtwVel) using speckle tracking echocardiography, where the ratio of PWV/GLS was used as a marker of ventricular-arterial interaction; and (e) Malondialdehyde (MDA) and protein carbonyls (PCs) plasma levels. Results: Intensified 12-month antidiabetic treatment reduced HbA1c (8.9 ± 1.8% (74 ± 24 mmol/mol) versus 7.1 ± 1.2% (54 ± 14 mmol/mol), *p* = 0.001), PWV (12 ± 3 versus 10.8 ± 2 m/s), PBR (2.12 ± 0.3 versus 1.98 ± 0.2 μm), MDA, and PCs; meanwhile, the treatment improved GLS (−15.2 versus −16.9%), PWV/GLS, and FMD% (*p* < 0.05). By multi-variate analysis, incretin-based agents were associated with improved PWV (*p* = 0.029), GLS (*p* = 0.037), PBR (*p* = 0.047), and FMD% (*p* = 0.034), in addition to a reduction of HbA1c. The patients with a final HbA1c ≤ 7% (≤ 53 mmol/mol) had greater reduction in PWV, PBR, and markers of oxidative stress, with a parallel increase in FMD and GLS, compared to those who had HbA1c > 7% (> 53 mmol/mol). Conclusions: Intensified glycaemic control, in addition to incretin-based treatment, improves arterial stiffness, endothelial glycocalyx, and myocardial deformation in type 2 diabetes after one year of treatment.

## 1. Introduction

Type 2 diabetes accelerates atherosclerosis and contributes to the development of diabetic cardiomyopathy [1,2]. Prospective epidemiologic studies have reported that the incidence and severity of these outcomes are strongly related to the severity of hyperglycemia [3]. However, the studies that examined the relationship between glycaemic control and cardiac function gave inconsistent results.

Markers of vascular function (namely, pulse wave velocity (PWV)) have been found to be elevated and flow-mediated dilation (FMD) to be decreased in type 2 diabetes [4]. Assessment of myocardial deformation of the left ventricle (LV) by speckle tracking echocardiography is superior to LV ejection fraction for the detection of early type 2 diabetic cardiac damage [5]. Elevated PWV has been associated to impaired longitudinal LV function in hypertensive patients [6]. The PWV to GLS ratio has been proposed as a marker of ventricular–arterial interaction [7].

The endothelial glycocalyx forms a layer of glycoproteins and proteoglycans that prevents direct contact of blood cells with the endothelial vascular cells [8]. Loss of glycocalyx integrity has been shown to occur after inflammatory or atherogenic stimuli, as well as during hyperglycemia. Damage of the glycocalyx results in enhanced sensitivity of the vasculature to atherogenic stimuli [9,10]. Novel imaging techniques, using dedicated cameras, have provided a non-invasive measurement of the glycocalyx thickness of the sublingual arterial micro-vessels [8,10].

However, it is not clear whether glycaemic control can lead to the improvement of arterial wall properties, endothelial glycocalyx, and decrease oxidative stress, which is a widely accepted participant in the development and progression of diabetes and its complications [11].

In the present study, we hypothesize that the intensification of glycaemic control, particularly by incretin-based treatment, may improve endothelial function, arterial elastic properties, LV myocardial strain, twisting–untwisting, and oxidative stress (as assessed by malondialdehyde (MDA) and protein carbonyls (PCs) blood levels).

## 2. Materials and Methods

### 2.1. Study Design

A total of 100 patients with type 2 diabetes were prospectively screened and recruited. Eligible patients included adults with type 2 diabetes with sub-optimal glycaemic control (glycated haemoglobin (HbA1c) ≥ 7% (≥ 53mmol/mol)). Patients were recruited consecutively from the Diabetes Centre outpatient clinic. Exclusion criteria were type 1 diabetes, history or clinical evidence of coronary artery disease, valvular heart disease, congestive heart failure, peripheral vascular disease, and liver or kidney failure. All subjects had normal resting electrocardiograms. Good glycaemic control was defined as HbA1c ≤ 7% (≤ 53 mmol/mol). We defined dyslipidaemia as total cholesterol > 220 mg/dL (low-density lipoprotein (LDL) > 100 mg/dL and/or high-density lipoprotein (HDL) < 40 mg/dL in men and HDL < 50 mg/dL in women) and/or triglycerides > 150 mg/dL, and hypertension as clinic blood pressure > 140/90 mmHg. The choice of the antidiabetic treatment was made according to the American Diabetes Association/European Association for the Study of Diabetes 2018 guidelines. We took into account cardiovascular risk, the presence of multiple risk factors of or established cardiovascular disease, the presence of excess weight, patient preferences (i.e., the fear of injections), fear of hypoglycaemia, and contra-indications (i.e., history of pancreatitis, gastroesophageal reflux disease, and so on). Thus, patients being overweight or obese, with non-alcoholic fatty liver disease (NAFLD), central obesity, dyslipidaemia, or hypertension were more likely to receive an incretin-based therapy. On the other hand, patients which presented catabolic symptoms, low weight, long-standing diabetes, or severe decompensation and profound hyperglycaemia were more likely to receive insulin.

The investigation conformed to the principles outlined in the Declaration of Helsinki. The study protocol was approved by the institute’s ethics committee, and written informed consents were obtained from all patients.

### 2.2. Primary and Secondary Endpoints

The primary endpoint was a change in arterial stiffness, as assessed by pulse wave velocity and augmentation index 12 months after intensified glycaemic control.

Secondary endpoints were changes in endothelial function, as assessed by flow-mediated dilation and glycocalyx thickness, LV myocardial deformation, and oxidative stress 12 months after glycaemic control.

### 2.3. Assessment of Arterial Stiffness

At baseline and after 12 months of intensive glycaemic control, we measured the carotid–femoral pulse wave velocity (PWV) and central aortic pressures using tonometry by Complior (Alam Medical, Vincennes, France). Normal values were PWV < 10 m/s [12]. Augmentation index (AI) was defined as 100 × (P2 − P1)/PP, where P2 is the late backward systolic wave, P1 is the early forward systolic wave, and PP is the pulse pressure, and represents the pressure boost that is induced by the return of the reflected waves at the aorta. AI_75_ was calculated to adjust the AI for a heart rate of 75 beats/min using the formula: AI_75_ = ((heart rate − 75) × 0.39) + AI [13]. Brachial blood pressure was measured in the right arm using an automated digital oscillometric sphygmomanometer (TensioMed Ltd., Budapest, Hungary).

### 2.4. Flow-Mediated Dilation

A linear array transducer (10 MHz) was used to measure the right brachial artery diameter at end diastole utilizing electronic calipers (Vivid E95 GE Medical Systems, Horten, Norway) and to record the baseline arterial blood flow velocity by color-coded Doppler ultrasonography (with an insonation angle of ≤60°). After a baseline assessment, a cuff fitted distally to the brachial artery was inflated (200–250 mmHg), altering arterial flow for 5 min. After cuff deflation, the hyperemic arterial blood flow velocity was recorded within the first 15 s and, afterwards, the brachial artery was scanned continuously for 90 s after cuff deflation, in order to define its maximal diameter (diameter during reactive hyperemia). Flow-mediated dilation (FMD) of the brachial artery was calculated as a percentage change of the arterial diameter after hyperemia from the baseline diameter [14]. Brachial artery flow was calculated according to the equation: Flow (mL/min) = ¼ mean flow velocity × heart rate × 3.14 (brachial artery diameter/2) We calculated the shear rate at rest and after hyperemia as an approximation of shear stress, averaged over the whole cardiac cycle by using the equation: Shear stress (in dyn/cm^2^) = 8 × μ × mean flow velocity/resting diameter, where μ is the viscosity of blood, as previously described [15]. The blood viscosity μ was assumed to be 0.035 dyne × s/cm^2^. All vasoactive medications (e.g., beta blockers, calcium channel blockers, angiotensin converting enzyme (ACE) inhibitors, or angiotensin II receptor blockers (ARB)) were discontinued for four half-lives of the drug before the vascular studies. Advice was given to keep a similar low-carbohydrate diet (specific for diabetics) throughout the study.

### 2.5. Endothelial Glycocalyx

We measured the perfused boundary region (PBR) of the sublingual arterial micro-vessels (which ranged from 5–25 μm) using side-stream dark-field imaging by dedicated cameras (Microscan, Glycocheck, Microvascular Health Solutions Inc., Salt Lake City, UT, USA). This method provides a non-invasive assessment of the endothelial glycocalyx [9]. The PBR is the cell-poor layer which results from the phase separation between the flowing red blood cell column and plasma on the surface of the vascular lumen. The PBR includes the luminal part of the glycocalyx that does permit cell penetration. Thus, an increased PBR indicates a deeper penetration of erythrocytes into the glycocalyx and is a marker of reduced glycocalyx thickness [9]. The measurement of glycocalyx thickness using dedicated cameras is easy to perform (duration of 3 min) and is operator-independent. Furthermore, it has a standardized methodology, provides measurements of >3000 vascular segments of the sublingual micro-vasculature, and has excellent reproducibility [9]. Thus, the technique was proposed for endothelial integrity assessment by the European Society of Cardiology Working Group on Peripheral Circulation [10].

### 2.6. Echocardiography

Studies were performed using a Vivid E95 (GE Medical Systems, Horten, Norway) ultrasound system. All studies were digitally stored in a computerized station (EchoPac GE 202, Horten, Norway) and were analyzed by two observers blind to the clinical and laboratory data.

### 2.7. Doppler Echocardiography

The E and A waves of the mitral inflow velocity were measured and the ratio E/A was calculated using pulse-wave Doppler [14].

### 2.8. 2D Strain and Strain Rate Analysis

Longitudinal systolic strain (LS) and systolic strain rate (LSR) were measured from 2-dimensional echocardiography images with a frame rate of 70–80 s^−1^, utilizing a dedicated software (EchoPac PC 202, GE Healthcare, Horten, Norway) [14]. We calculated the global longitudinal strain (GLS) and global longitudinal strain rate (GLSR) using the 17 LV myocardial segment model, imaged using the apical 4-, 2-, and 3-chamber views [13]. The normal value for GLS was −20% [16].

### 2.9. Ventricular-Arterial Interaction

The ratio of carotid–femoral PWV to global longitudinal strain (m/s−%) was calculated as an index of ventricular–arterial interaction, as previously published [7,17]. The ratio had negative values, in accordance with the negative GLS values; thus, the more negative the value, the less abnormal.

### 2.10. LV Twisting and Untwisting

The parasternal short axis views at basal and apical level were used to assess LV twisting and untwisting [14]. Twisting–untwisting rotation and velocity curves along time were constructed using a commercially available software (EchoPac PC 202, GE Healthcare). From the mitral valve inflow Doppler recording, we measured the time interval between the onset of the QRS interval of the ECG trace and the onset, peak, and end of the mitral E waveform [14]. Utilizing the above-measured time intervals, we estimated peak twisting (pTw, deg), as well as untwisting, at the time of mitral valve opening (Utw_MVO_), peak (Utw_PEF_), and at the end of left ventricular early filling (Utw_EDF_). LV untwisting during diastole was estimated as the percentage difference between peak twisting and untwisting at MVO (%dpTw-Utw_MVO_), at the peak (%dpTw-Utw_PEF_), and at the end of early filling (%dpTw-Utw_EDF_) [14]. Furthermore, peak twisting (pTw, deg), peak twisting velocity (pTwVel, deg/s), and peak untwisting velocity (pUtwVel, deg/s) were measured by the respective rotation curves.

### 2.11. Laboratory Assays

We measured malondialdehyde (MDA) and protein carbonyls (PCs) spectrophotometrically (Oxford Biomedical Research, Rochester Hills, MI) using a colorimetric assay for lipid peroxidation (measurement range 1–20 nmol/L) and assessment of the 2,4-dinitrophenylhydrazine derivatives of protein carbonyls (nmol/mg protein), respectively [13,18].

### 2.12. Statistical Analysis

The SPSS 21.0 statistical software package was used for analysis. We compared the categorical variables by a standard chi-square test. We used the Kolmogorov–Smirnov test to examine the distribution of continuous variables. Normally distributed variables are expressed as mean ± standard deviation. Data with a non-Gaussian distribution are shown as median (interquartile range) and transformed into ranks for analysis. A t-test (or paired t-test) was used to compare differences in mean values of continuous variables. The Mann–Whitney U-test (or Wilcoxon signed-rank test) was applied for non-normally distributed variables. Cross-sectional associations were examined by parametric (Pearson r) or non-parametric (Spearman rho) correlation coefficients. Comparison of the measurements of the examined markers at baseline and 12 months after glycaemic control was performed by ANOVA, with adjustment for age, sex, and interactions. Post-hoc comparisons were performed by Bonferroni correction.

Linear regression was used to investigate relations between improvement in GLS, PWV, PBR, and FMD with changes in HbA1c. Multiple linear regression was used to identify independent predictors of GLS, PWV, PBR, and FMD at follow-up and changes in these outcomes (Δ parameters) from their respective baseline values. Parameters with *p* < 0.1 on univariable analysis were entered into the final multi-variable model as explanatory variables. Independent contributions were then assessed, and there was no evidence of significant multi-collinearity. The anthropometric and metabolic values at the 12-month follow-up were used for predicting GLS, PWV, PBR, and FMD on follow-up. Changes between baseline and 12-month follow-up (Δ parameters) for the continuous variables were used to predict ΔGLS and ΔPWV.

## 3. Results

### 3.1. Study Population

The 100 patients (65 men and 35 women) had a mean age of 51 ± 12 years, and a mean duration of type 2 diabetes of 3.2 ± 1.5 years. Their baseline and 12-month follow-up clinical and metabolic characteristics are presented in Table 1. Patients had poorly controlled diabetes: A total of 13 patients had HbA1c < 8.0% (<64 mmol/mol), 44 had HbA1c between 8.0–9.0% (64–75 mmol/mol), 23 patients had HbA1c between 9.0–10.0% (75–86 mmol/mol), and 20 patients had HbA1c > 10.0% (>86 mmol/mol). There were no differences in cardiovascular medication between patients with HbA1c ≤ 7% and > 7% at the 1 year follow up (*p* > 0.05). In particular, 58% of the patients with HbA1c ≤ 7% and 42% with HbA1c > 7% were treated with statins (*p* = 0.76). Age was similar between patients with HbA1 < 7% and > 7% (53 ± 10 versus 50 ± 15 years, *p* = 0.12). The smoking status remained unchanged in the smoker patients at 1 year post treatment.

### 3.2. Interrelation between Metabolic, Endothelial, Vascular, and LV Function Markers at Baseline

At baseline, HbA1c was positively associated with PBR 20–25 μm (r = 0.43, *p* = 0.012) and negatively related with FMD% (r = −0.30, *p* = 0.010). Furthermore, HbA1c was related with GLS (r=0.38, p=0.012) and peak twisting (r = 0.30, *p* = 0.035), and reversely associated with %dpTw-Utw_MVO_ (r = −0.35, *p* = 0.032). Additionally, HbA1c was correlated with the PWV/GLS ratio and MDA levels (r = 0.30, *p* = 0.021 and r = 0.34, *p* = 0.032, respectively).

MDA and PCs were related with PWV (r = 0.35, *p* = 0.022 and r = 0.26, *p* = 0.045, respectively). Moreover, PCs were reversely associated with GLSR E (r = −0.37, *p* = 0.03) and positively related with PBR 5–25 μm (r = 0.40, *p* = 0.046).

PWV was correlated with GLS (r = 0.46, *p* < 0.001) and reversely associated with GLSR E (r = −0.40, *p* < 0.001). Furthermore, PWV was positively related with peak twisting (r = 0.46, *p* = 0.028).

### 3.3. Changes in Metabolic Parameters after 12-Month Glycaemic Control

HbA1c improved significantly post treatment (Table 1, *p* = 0.001). There were no significant changes in BMI, waist circumference, and lipid profile in the overall population. However, patients on incretin-based agents showed a significant reduction of BMI (32 ± 4 versus 27 ± 6 kg/m^2^, *p* = 0.03).

### 3.4. Effect of Glycaemic Control on Vascular and Endothelial Function

Table 2 shows the baseline and 12-month follow-up characteristics of the patients. Furthermore, the follow-up parameters are divided according to whether patients achieved HbA1c ≤ 7% (≤ 53 mmol/mol) or > 7% (> 53 mmol/mol).

**Figure 1:**
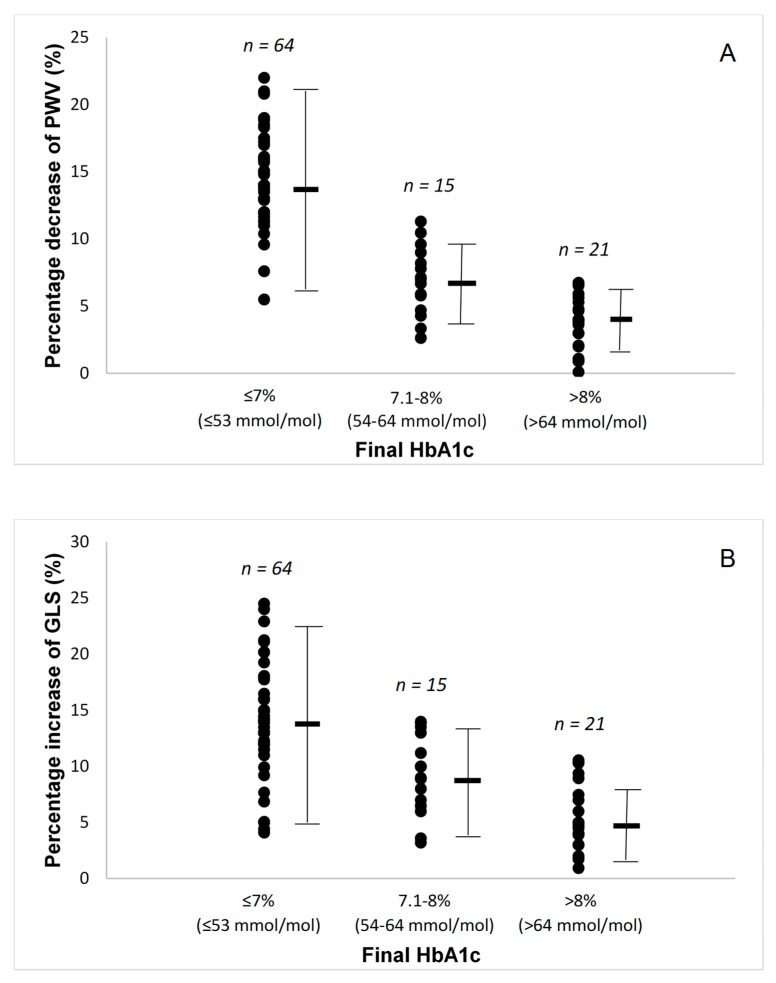

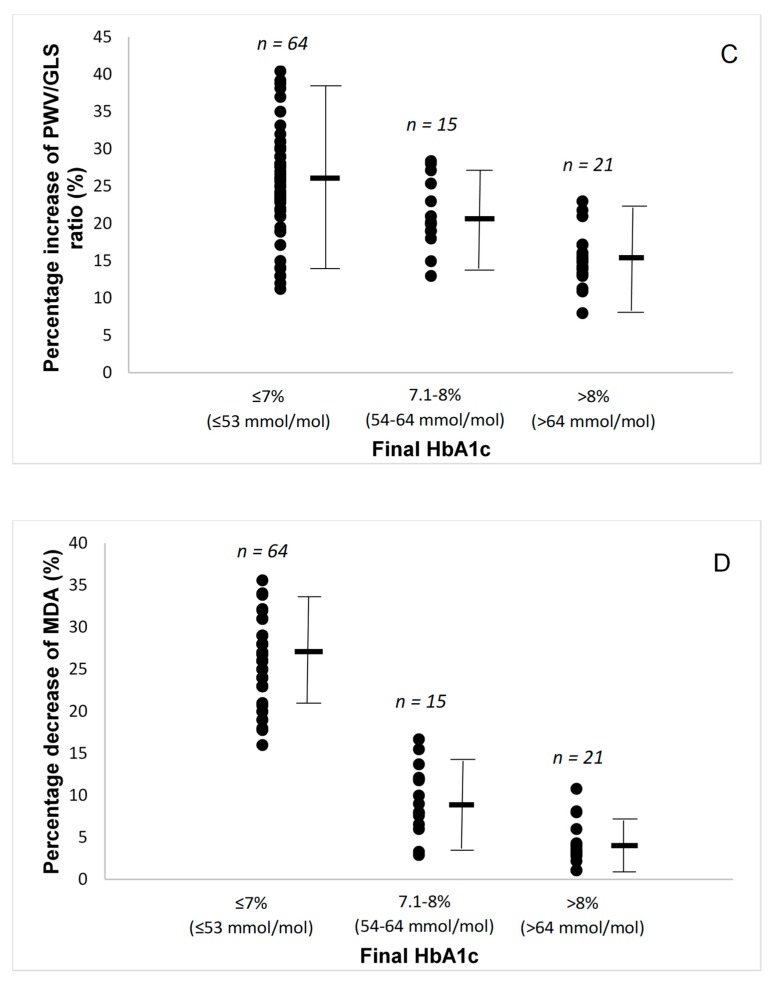

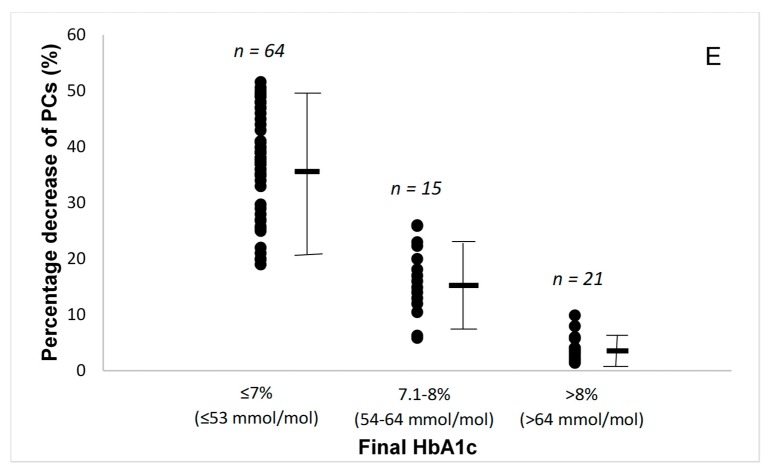
(**A**) Percentage changes in pulse wave velocity (PWV) by the final glycated haemoglobin (HbA1c) attained. (**B**) Percentage changes in global longitudinal strain (GLS) by the final glycated haemoglobin (HbA1c) attained. (**C**) Percentage changes in pulse wave velocity to global longitudinal strain ratio by the final glycated haemoglobin (HbA1c) attained. (**D**) Percentage changes in malondialdehyde (MDA) by the final glycated haemoglobin (HbA1c) attained. (**E**) Percentage changes in protein carbonyls (PCs) by the final glycated haemoglobin (HbA1c) attained. Lines are mean and SD.

In all patients, there was reduction in brachial and central systolic blood pressure (Table 1) with a parallel reduction in arterial stiffness, as assessed by PWV and AI (Table 2). In addition, both FMD and PBR 5–25 μm improved. No significant changes were observed in resting or hyperemic brachial arterial blood flow, velocities, and shear rate at 12 months post treatment, compared to baseline (*p* > 0.05). There was a significantly greater improvement in PWV (Figure 1A), FMD, and PBR in patients who achieved HbA1c ≤ 7% (≤ 53 mmol/mol) (Table 2).

### 3.5. Speckle Tracking Analysis

#### 3.5.1. Longitudinal Strain

Baseline echocardiographic parameters are presented in Table 2. Patients had normal ejection fraction but low mean value of GLS, indicating impaired systolic function. Compared with baseline, all patients had improved GLS and strain rate S (*p* < 0.01), as well as improved strain rate E at early diastole and reduced LA volume after a 12-month period, suggesting improved systolic and diastolic function (*p* < 0.05). There was a progressively greater improvement in GLS, GLSrE, LA volume, and PWV/GLS ratio as the patients achieved a lower final HbA1_C_, with the patients with final HbA1c ≤ 7% (≤53 mmol/mol) having the largest improvements (Figure 1B,C).

#### 3.5.2. LV Twisting and Untwisting Velocity

Compared to baseline, after 12 months of glycaemic control, all patients showed a significant decrease in peak twisting and increase in peak untwisting velocity (*p* < 0.05). In addition, the percentage changes between peak twisting and untwisting at mitral valve opening (%dpTw-Utw_MVO_) and peak (%dpTw-Utw_PEF_) of early diastolic LV filling were increased (*p* < 0.05, Table 2), suggesting a beneficial effect of good glycaemic control on LV twisting–untwisting.

### 3.6. Markers of Oxidative Stress

During the 12-month study period, all patients achieved a reduction of oxidative stress burden, as assessed by malondialdehyde (MDA) and protein carbonyls (PCs) concentrations (Table 2). The patients with a final HbA1c ≤ 7% (≤ 53 mmol/mol) had significantly greater reduction in these markers of oxidative stress (Figure 1D,E).

### 3.7. Interrelation between Metabolic, Endothelial, Vascular, and LV Function Markers after 12-Month Glycaemic Control

After 12 months, the percentage reduction of HbA1c was related with the percentage decrease of PBR 5–25 μm (r = 0.46, *p* = 0.043) and the percentage increases of GLS and GLSRE (r = 0.46, *p* = 0.006 and r = −0.45, *p* = 0.044, respectively). Furthermore, the percentage improvement of HbA1c was significantly correlated with the percentage reduction of PWV and PWV/GLS ratio (r = 0.43, *p* = 0.012 and r = 0.56, *p* = 0.013, respectively) after the 12-month follow-up. Furthermore, the percentage reduction of HbA1c was related with the reduction of MDA and PCs (r = 0.42, *p* = 0.039 and r = 0.94, *p* = 0.020, respectively).

The percentage improvement of MDA and PCs was significantly correlated with the percentage reduction of PWV (r = 0.57, *p* = 0.018 and r = 0.62, 0.010, respectively). Furthermore, the reduction of MDA was positively associated with the reduction of cSBP (r = 0.55, *p* = 0.020). Moreover, the reduction of MDA was negatively correlated with increase of FMD% and %dpTw-Utw_PEF_ (r = −0.88, *p* = 0.041 and r = −0.54, *p* = 0.026, respectively). The percentage reduction of PCs was correlated with the percentage increase of GLS after the 12-month follow-up (r = 0.50, *p* = 0.021).

The percentage reduction of PBR 5–25 μm was negatively related with the percentage increase of FMD and GLS (r = −0.35, *p* = 0.007 and r = −0.43, *p* = 0.015, respectively). Moreover, the percentage reduction of PBR 5–25 μm was positively correlated with the PWV/GLS ratio (r = 0.44, *p* = 0.012) at follow-up.

Lastly, the percentage reduction of PWV was significantly correlated with the percentage improvement of GLS and the percentage difference between peak twisting and untwisting at peak of early LV diastolic filling (%dpTw-Utw_PEF_) (r = 0.39, *p* = 0.024 and r = 0.37, *p* = 0.043, respectively).

By multi-variate analysis, treatment with DDP-4 inhibitors and GLP-1 receptor agonists (incretin-based agents) were associated with improved PWV, GLS, and PBR, in addition to HbA1 value changes. Duration of type 2 diabetes, history of hypertension, baseline value, and change in HbA1c were independent predictors of change in GLS. Age, smoking, and change in HbA1c were independent predictors of change in PWV. Furthermore, BMI, history of hypertension and dyslipidemia, change in HbA1c, and treatment with basal insulin analogs were independent predictors of change in PBR. Additionally, independent predictors of change in FMD were age, duration of type 2 diabetes, treatment with basal insulin analogs, and treatment with DDP-4 inhibitors and GLP-1 receptor agonists (incretin-based agents), but not the change in HbA1c (*p* > 0.05) after 1 year of treatment (Table 3). There were no sex-specific differences in the changes of the vascular and cardiac function markers, post treatment.

## 4. Discussion

In the present study, intensifying glycaemic control over a 12-month period led to a significant reduction of arterial stiffness and improvement of endothelial function, as assessed by FMD and endothelial glycocalyx thickness, in parallel with an improvement of LV myocardial strain and twisting–untwisting and reduction of oxidative stress burden. Additionally, the reduction of oxidative stress burden was related with improved arterial elastic properties, as assessed by PWV; which, in turn, was related with improvement of LV myocardial deformation after optimizing glycaemic control. Incretin-based treatment (GLP-1 and DPP-4) was associated with the improvement of cardiovascular function markers, in addition to effective glycaemic control, as assessed by HbA1 value changes.

### 4.1. Impact of Glycaemic Control on Vascular and Endothelial Function

Type 2 diabetes has been associated with increased arterial [4] and endothelial dysfunction [19]. A recent study demonstrated that good glycaemic control in patients with type 2 diabetes and cardiovascular disease was associated with better micro-vascular function, whereas, in those with advanced disease, this association was lost [20]. The mechanisms by which hyperglycemia causes arterial stiffening are not clear. However, it is likely that increased glucose levels contribute to an imbalance between the protective versus the detrimental pathways, such as enhancement of the sorvitol, protein kinase C, and pentose phosphate pathways. These pathways are detrimental to the vascular endothelial function, as they lead to increased oxidative stress and accelerated apoptosis of endothelial cells. Furthermore, the availability of NO is also reduced during hyperglycemia, resulting in profound endothelial dysfunction [21]. On the other hand, Yue et al [22] have shown that the degree of glycaemic control in patients with type 2 diabetes was linked to the levels of circulating endothelial progenitor cells (EPCs), as well as the arterial stiffness, and that diabetic patients who showed an adequate glycaemic control had a higher circulating EPC count and lower arterial stiffness. In agreement with the above, our study demonstrated that better glycaemic control was associated with reduced arterial stiffness and central aortic pressure, as well as improved endothelial glycocalyx integrity, in subjects with type 2 diabetes. Furthermore, treatment with incretin-based agents had incremental predictive value in the improvement of PWV, in addition to HbA1 value changes, expanding the findings of our previous study with liraglutide [23]. Moreover, in the present study, a 12-month treatment with insulin was associated with improvement in FMD at follow-up, suggesting a beneficial effect of insulin on vascular function. Finally, independent to HbA1 changes, association of incretin-based treatment with improved FMD suggests that this beneficial response of endothelial function may be attributed to the pleiotropic effects of incretin-based treatment.

Acute and long-term hyperglycemia, oxidative stress, and inflammation have been associated with the loss of glycocalyx integrity [8,24]. In our previous study, we demonstrated that first-degree relatives of diabetic patients and dysglycaemic subjects who had higher insulin and glucose levels, also had higher baseline values of PBR than normoglycaemics [25]. Actually, in the present study, we report for the first time that HbA1c was positively correlated with PBR, suggesting that excessive hyperglycemia may contribute to the loss of glycocalyx integrity. A perturbed glycocalyx facilitates attachment and rolling of circulating inflammatory cells in the sub-endothelial layers and increases endothelial permeability to oxidized lipids and proteins [26]. Thus, glycocalyx damage may promote inflammation, oxidative stress, and apoptosis within the myocardial layers [24,26]. Indeed, in the present study, we showed that the endothelial glycocalyx improved after successful glycaemic control and that the latter was associated with the improvement of oxidative stress markers after 12 months of intensive antidiabetic treatment. Finally, the change of BMI was independently associated with the respective improvement of PWV and endothelial glycocalyx, suggesting that weight reduction may be an additional mechanism mediating the beneficial effects of intensive antidiabetic treatment. Similarly to the FMD improvement, treatment with incretin-based agents was related with the respective increase of glycocalyx thickness at the 1 year follow-up, in addition to effective reduction of HbA1 value.

### 4.2. Effect of Glycaemic Control on LV Myocardial Deformation

Asymptomatic diabetic patients manifest complications that are closely associated with systolic dysfunction [27], despite a preserved LV ejection fraction. The underlying pathophysiologic mechanisms of diabetic cardiomyopathy are multi-factorial, where hyperglycaemia plays a central role [2]. However, the evidence to date on the relationship between glycaemic control and myocardial function has been contradictory. Some studies have shown that poor glycaemic control was associated with abnormal LV relaxation and lower systolic strain [28,29]. However, other studies have demonstrated no significant association between glycaemic control and cardiac function [30,31,32,33]. Several studies failed to demonstrate improvement in either diastolic or systolic function, despite better glycaemic control [34,35]. On the other hand, a recent study demonstrated that both the degree of improvement when compared with baseline and the final glycaemic status over a 12-month period determined the improvement in LV systolic and diastolic function [36].

Arterial stiffness is one of the determinants of LV longitudinal strain and twisting–untwisting by affecting perfusion of sub-endocardial myocardial fibers [14]. Furthermore, arterial wave reflections have been linked to LV diastolic dysfunction [37]. Moreover, the PWV/GLS ratio, a novel marker of ventricular–arterial interaction [7], has been related to impaired diastolic function in hypertension [17]. Ιn our study, the reduced brachial and central systolic blood pressure and decreased arterial stiffness indicated a significant afterload reduction, which may have contributed to the improvement of myocardial perfusion and, consequently, to improved LV function and ventricular–arterial interaction, as assessed by the PWV/GLS ratio [7,14,17]. Indeed, lower values of PWV after the significant improvement of glycaemic control has been related to the respective improvements of GLS and GLSR. Additionally, in our study, the improved longitudinal deformation post treatment led to the normalization of an abnormally augmented LV twisting before treatment. Furthermore, treatment with incretin-based agents was an independent predictor of change in longitudinal strain, in addition to effective reduction of HbA1 value, expanding on the findings of our previous study [23]. Indeed, incretin-based agents appear to produce favorable cardiovascular effects among individuals with diabetes and may reduce the risk of heart failure events [38].

### 4.3. Glycaemic Control and Oxidative Stress

Lipid peroxidation and protein oxidation are commonly assessed by malondialdehyde and PCs levels, respectively [39]. Glycation in diabetes has been found to induce the formation of protein carbonyls, thus generating reactive radicals and perpetuating a vicious cycle [40]. Oxidative stress has a direct negative inotropic effect on the myocardium and promotes myocardial ischemia [14]. Hence, oxidative stress-induced vascular dysfunction may explain the link between arterial stiffening and impaired LV myocardial deformation [41]. The interactions between oxidative stress, inflammation, and endothelial dysfunction in type 2 diabetes have been previous reported [42]. A previous study of ours showed that a 6-month treatment with a GLP-1 analogue improved arterial elasticity, LV myocardial strain, and twisting and untwisting by reduction of oxidative stress burden in type 2 diabetes [23]. In this study, baseline PCs levels determined impaired endothelial glycocalyx thickness. Furthermore, the reduction of oxidative stress was associated with improved arterial elasticity and myocardial deformation after 12 months of intensive antidiabetic treatment. The decrease of MDA and PCs was greater in patients that achieved HbA1c ≤ 7% (≤53 mmol/mol) than those who had final HbA1c > 7% (>53 mmol/mol). Thus, a decrease of oxidative stress burden was linked with a greater improvement of vascular function and myocardial deformation.

In our previous study, oxidative stress was reduced after 6 months of treatment with a GLP-1 analogue, while this improvement was not evident in the group treated with metformin, despite a similar change of HbA1 between the two treatment arms, suggesting an early improvement of oxidative stress by incretin-based treatment. In the current study, after 1 year of treatment both change of HbA1 and incretin-based treatment are independent determinants of improved GLS and PWV. Moreover, in this study, the percentage reduction of HbA1c was related with the reduction of MDA and PCs after 1 year of antidiabetic treatment; furthermore, the reduction of oxidative stress was associated with improved PWV and myocardial deformation after 1 year of treatment. Thus, incretin-based treatment for a longer period contributes to an optimal glucose control, as assessed by HbA1 changes, and thus to a further reduction of oxidative stress, resulting in a further improvement of vascular and myocardial function than the shorter term treatment period (6 months) used in our previous study.

### 4.4. Study Limitations

The present study was conducted as a single-arm interventional study, with each patient acting as their own control, and we compared those who improved glycaemic control with those who did not. Moreover, the effects of improved glycaemic control on endothelial and myocardial function observed in this study are only preliminary. Large-scale studies are needed to confirm the findings.

## 5. Conclusions

Intensified glycaemic control over a 12-month period improved arterial stiffness, LV myocardial deformation, and, consequently, ventricular–arterial interaction, probably by reduction of oxidative stress and endothelial dysfunction, in initially poorly controlled type 2 diabetic patients. Glycaemic control may reverse early subclinical cardiac and vascular dysfunction and reduce the risk of heart failure. Incretin-based treatment has an additive value to effective glycaemic control, as assessed by HbA1c value, for the improvement of cardiovascular function after 1 year of treatment. Thus, incretin-based agents appear to produce favorable cardiovascular effects among individuals with diabetes and may reduce the risk of heart failure events.

## Figures and Tables

**Table 1:** Clinical and metabolic characteristics of patients.

	Baseline (*n* = 100)	12 months (*n* = 100)	*p*
Clinical features			
Weight, kg	88 ± 16	86 ± 15	0.560
BMI, kg/m^2^	31 ± 5	29 ± 6	0.167
Waist, cm	104 ± 13	102 ± 14	0.421
SBP, mmHg	138 ± 17	134 ± 16	0.042
DBP, mmHg	86 ± 11	84 ± 11	0.093
cSBP, mmHg	139 ± 20	134 ± 16	0.039
Metabolic characteristics			
HbA1c, % (mmol/mol)	8.9 ± 1.8 (74 ± 24)	7.1 ± 1.2 (54 ± 14)	0.001
Fasting glucose, mg/dL	165 ± 47	124 ± 40	0.007
Total cholesterol, mg/dL	190 ± 34	186 ± 31	0.402
LDL cholesterol, mg/dL	116 ± 32	109 ± 29	0.244
HDL cholesterol, mg/dL	47 ± 10	52 ± 12	0.318
Triglycerides, mg/dL	167 ± 92	159 ± 86	0.286
Creatinine, mmol/L	1.1 ± 0.2	1.0 ± 0.2	0.734
eGFR, mL/min per 1.73 m^2^	85 ± 8	86 ± 9	0.321
Cardiac risk factors			
Smoking, % (*n*)	37 (37)	34 (34)	0.675
Hypertension, % (*n*)	50 (50)	47 (47)	0.277
Dyslipidemia, % (*n*)	51 (51)	48 (48)	0.243
Family history CAD, % (*n*)	21 (21)	21 (21)	0.889
Cardiovascular medication, % (*n*)			
Aspirin or clopidogrel	8 (8)	8 (8)	0.910
Beta blockers	19 (19)	21 (21)	0.753
Calcium channel blocker	30 (30)	33 (33)	0.631
ACE inhibitor or ARB	33 (33)	36 (36)	0.270
Diuretics	14 (14)	15 (15)	0.798
Statins	46 (46)	49 (49)	0.610
Fibrate	5 (5)	8 (8)	0.270
Antidiabetic medication, % (n)			
Metformin	85 (85)	90 (90)	
Sulfonylureas	4 (4)	5 (5)	
Incretin-based agents	0 (0)	78 (78)	
DPP-4 inhibitors	0 (0)	38 (38)	
GLP-1 receptor agonists	0 (0)	40 (40)	
Basal insulin analogs	25 (25)	39 (39)	
Rapid-acting insulin analogs	5 (5)	7 (7)	
Glucose-lowering agents used for intensified antidiabetic treatment, % (*n*)			
Metformin		5 (5)	
Sulfonylureas		1 (1)	
Incretin-based agents		78 (78)	
DPP-4 inhibitors		38 (38)	
GLP-1 receptor agonists		40 (40)	
Basal insulin analogs		14 (14)	
Rapid-acting insulin analogs		2 (2)	

Data are expressed as the mean (SD) or *n* (%). Continuous variables were compared with the paired Student t-test. Binary variables were compared with the χ^2^ test. Ordinal variables were compared with the Wilcoxon signed-rank or Wilcoxon rank-sum test, where appropriate. BMI, body mass index; SBP, systolic blood pressure; DBP, diastolic blood pressure; cSBP, central systolic blood pressure; HbA1c, glycated haemoglobin; LDL, low-density lipoprotein; HDL, high-density lipoprotein; eGFR, estimated glomerular filtration rate; CAD, coronary artery disease; Incretin-based agents: DPP-4, Dipeptidyl peptidase-4 inhibitors and GLP-1, Glucagon-like peptide-1 receptor agonists.

**Table 2:** Baseline and follow-up values of metabolic characteristics, arterial stiffness, endothelial function, LV function, and oxidative stress.

Characteristics	Baseline	Follow-up				
	(*n* = 100)	(*n* = 100)	HbA1c ≤ 7% (≤53 mmol/mol) (*n* = 64)	Δ% (*n* = 64)	HbA1c >7% (>53 mmol/mol) (*n* = 36)	Δ% (*n* = 36)
PWV, m/s	12 ± 3	10.8 ± 2 ***	10.5 ± 2	−13.9	11.2 ± 2 †	−5.1
AI, %	17 (−0.5–30)	11.5 (1–25) **	11.5 (1–25)	−32	10.5 (0.4–23)	−38
AI_75_, %	16 (−1–28)	11 (0.5–24) **	11 (0.5–24)	−31	10 (0.34–22)	−37.5
HR, bpm	73 ± 11	74 ± 10	75 ± 10	2.7	73 ± 10	0
SBP, mmHg	138 ± 17	134 ± 16 *	133 ± 17	−3.6	135 ± 14	−2.2
DBP, mmHg	86 ± 11	84 ± 11	84 ± 10	−2.3	85 ± 12	−1.2
cSBP, mmHg	139 ± 20	134 ± 16 *	132 ± 17	−5.7	134 ± 15	−2.9
FMD%	8.1 ± 5	11.7 ± 8 **	14 ± 8	68.7	9.5 ± 7 †	20
PBR, 5–25 μm	2.12 ± 0.3	1.98 ± 0.2 *	1.88 ± 0.2	−10.9	2.01 ± 0.3 †	−5.6
PBR, 5–9 μm	1.19 ± 0.1	1.14 ± 0.1	1.08 ± 0.2	−9.2	1.12 ± 0.2	−5.9
PBR, 10–19 μm	2.26 ± 0.3	2.08 ± 0.3 *	1.97 ± 0.3	−13.2	2.10 ± 0.3 †	−6.6
PBR, 20–25 μm	2.63 ± 0.5	2.46 ± 0.4 *	2.35 ± 0.3	−11	2.50 ± 0.5 †	−4.6
LVEF, %	65 ± 10	67 ± 9	68 ± 9	4.6	67 ± 6	3
GLS, %	−15.2 ± 3	−16.9 ± 3 **	−17.6 ± 3	14.3	−16 ± 3 ††	6.7
GLSR S, 1/s	−0.78 ± 0.2	−0.88 ± 0.2 **	−0.91 ± 0.2	15	−0.86 ± 0.2 †	11.4
GLSR E, 1/s	0.82 ± 0.3	0.96 ± 0.3 **	1.04 ± 0.4	28.4	0.87 ± 0.2 ††	4.8
GLSR A, 1/s	0.72 ± 0.3	0.75 ± 0.2	0.82 ± 0.2	13.8	0.70 ± 0.2 ††	3
PWV/GLS	−0.84 ± 0.3	−0.66 ± 0.2 ***	−0.62 ± 0.2	−26.2	−0.69 ± 0.2	−17.9
pTw, deg	16 ± 5	14.9 ± 7 **	15.4 ± 5	−4.3	14.5 ± 8	−8.8
pUtwVel, deg/s	−95 ± 45	−109 ± 50 *	−112 ± 44	16.7	−98 ± 49 †	4.3
%dpTw-Utw_MVO_	28 ± 9	37 ± 11 *	38 ± 14	35.7	37 ± 13	32
%dpTw-Utw_PEF_	46 ± 19	59 ± 18 *	61 ± 20	29.8	58 ± 21	28.9
LA volume (mL/m^2^)	40 ± 3	33 ± 2 *	32 ± 2	−20.1	38 ± 21 ††	−5.1
E/A	0.95 ± 0.4	0.98 ± 0.3	1.04 ± 0.3	8.3	0.94 ± 0.3	−2.1
E/e’	7.7 ± 1.8	7.8 ± 2.4	8.2 ± 2.3	9.3	7.4 ± 1.7	−1.3
MDA, nM/L	0.95 (0.56–1.7)	0.75 * (0.50–1.5)	0.71 (0.45–1.28)	−26.8	0.83 † (0.48–1.58)	−10.8
PCs, nmol/mg protein	0.016 (0.008–0.021)	0.013 * (0.009–0.016)	0.010 (0.009–0.016)	−37.5	0.013 † (0.01–0.018)	−18.8

Data are presented as mean ± SD values. Δ%: percent changes between baseline and 12-month follow-up. Values for AI, AI_75_, and biomarkers are median and interquartile range. HbA1c, glycated haemoglobin A1c; PWV, pulse wave velocity; AI_75_ = ((heart rate–75) × 0.39) + AI was calculated to adjust the AI for a heart rate of 75 beats/min; cSBP, central systolic blood pressure; FMD%, percentage difference of flow-mediated dilatation; PBR, perfused boundary region of the sublingual arterial micro-vessels ranging from 5–25 μm; LVEF, left ventricular ejection fraction; GLS, global longitudinal strain; GLSR, global londitudinal strain rate; PWV/GLS, ventricular–arterial interaction (pulse wave velocity to global longitudinal strain ratio); pTw, peak twisting; pUtw velocity, peak untwisting velocity; %dpTw-Utw_MVO_, percentage difference between peak twisting and untwisting at mitral valve opening (MVO); %dpTw-Utw_PEF_, percentage difference between peak twisting and untwisting at peak of left ventricular early filling; E/A ratio, ratio of E to A waves of the mitral inflow velocity; MDA, malondialdehyde; PCs, protein carbonyls. * *p* < 0.05; ** *p* < 0.01; *** *p* < 0.001 versus baseline; † *p* < 0.05; †† *p* < 0.01 versus subgroup with HbA1c ≤ 7% (≤ 53 mmol/mol).

**Table 3:** Association of the changes of LV myocardial strain, as well as arterial and endothelial markers, with parameters of the study population.

**ΔGLS**			
Univariate			
	**Unstandardized b**	**95% CI**	***p* value**
**Age**	−0.292	−0.815–0.231	0.061
**BMI**	−0.089	−0.224–0.087	0.039
**Duration of diabetes**	−0.889	−2.455–0.677	0.023
**Smoking**	−1.380	−2.752–0.008	0.049
**Hypertension**	−3.170	−8.095–1.755	0.017
**HbA1c**	−0.724	−1.358–0.331	0.009
**ΔHbA1c**	0.923	−0.795–1.720	0.033
**Incretin-based agents**	1.178	−0.811–2.348	0.011
Multivariate			
**Duration of diabetes**	−0.209	−0.756–0.343	0.048
**Hypertension**	−2.155	−3.545–1.942	0.046
**HbA1c**	−0.523	−1.576–1.190	0.039
**ΔHbA1c**	0.876	−1.109–2.141	0.041
**Incretin-based agents**	0.687	−3.518–5.269	0.037
**ΔPWV**			
Univariate			
	**Unstandardized b**	**95% CI**	***p* value**
**Age**	−0.880	−1.297–0.172	0.018
**BMI**	−0.061	−0.179–0.055	0.070
**Smoking**	−1.438	−3.608–0.731	0.017
**ΔHbA1c**	0.562	−0.133–0.992	0.004
**Incretin-based agents**	2.244	−0.872–3.617	0.002
Multivariate			
**Age**	−0.435	−0.423–0.038	0.021
**Smoking**	−1.203	−2.443–0.085	0.036
**ΔHbA1c**	0.447	−0.064–1.088	0.025
**Incretin-based agents**	1.237	−0.215–2.514	0.029
**ΔPBR**			
Univariate			
	**Unstandardized b**	**95% CI**	***p* value**
**BMI**	−0.058	−0.054–0.170	0.009
**Duration of diabetes**	−0.188	−0.449–0.073	0.093
**Hypertension**	−0.603	−0.764–0.178	0.033
**Dyslipidemia**	−0.831	−1.010–0.147	0.030
**ΔHbA1c**	0.650	0.224–0.997	0.016
**Incretin-based agents**	0.687	0.305–1.035	0.037
**Basal insulin analogs**	0.864	−1.183–2.519	0.010
Multivariate			
**BMI**	−0.031	−0.059–−0.021	0.041
**Hypertension**	−0.427	−0.837–−0.018	0.044
**Dyslipidemia**	−0.245	−1.804–0.343	0.045
**ΔHbA1c**	0.395	−0.039–0.772	0.038
**Incretin-based agents**	0.429	−0.029–0.787	0.047
**Basal insulin analog**	0.628	0.539–1.918	0.042
**ΔFMD**%			
Univariate			
	**Unstandardized b**	**95% CI**	***p* value**
**Age**	−0.417	−1.502–0.919	0.034
**Duration of diabetes**	−2.269	−4.565–0.027	0.017
**HbA1c**	−0.171	−2.043–1.701	0.084
**Incretin-based agents**	1.491	−1.953–4.253	0.014
**Basal insulin analogs**	1.092	0.345–2.495	0.013
*Multivariate*			
**Age**	−0.399	−0.432–0.012	0.046
**Duration of diabetes**	−0.980	−2.135–0.532	0.029
**Incretin-based agents**	1.159	0.913–2.384	0.034
**Basal insulin analogs**	0.894	0.574–1.898	0.046

Δ: change between baseline and 12-month follow-up. GLS, global longitudinal strain; PWV, pulse wave velocity; PBR, perfused boundary region of the sublingual arterial micro-vessels ranging from 5–25 μm; FMD, flow-mediaded dilatation of the brachial artery; BMI, body mass index; HbA1c, glycated haemoglobin A1c; Incretin-based agents: DPP-4, Dipeptidyl peptidase-4 inhibitors and GLP-1, Glucagon-like peptide-1 receptor agonists. The HbA1c value refers to baseline.
